# Temporomandibular joint arthrocententesis: evaluation of results and review of the literature

**DOI:** 10.1016/S1808-8694(15)31019-3

**Published:** 2015-10-19

**Authors:** Belmiro Cavalcanti do Egito Vasconcelos, Ricardo Viana Bessa-Nogueira, Nelson Studart Rocha

**Affiliations:** a PhD, Post-Graduation Program Coordinator - UPE.; b Bucco-maxillofacial traumatology and surgery (BMFTS) Specialist and MS – Dentistry School - UPE. PhD student in CTBMF / Pernambuco Dentistry School - UPE.; c Specialist in Bucco-maxillofacial traumatology and surgery (BMFTS) – Dentistry School of Pernambuco (FOP/UPE). Bucco-maxillofacial surgeon - Getulio Vargas Hospital-Recife/PE. University of Pernambuco – School of Dentistry – MS and PhD programs in Bucco-maxillofacial traumatology and surgery (BMFTS).

**Keywords:** Arthrocentesis, temporomandibular joint

## Abstract

**Aim:**

This study was designed to investigate the effects of arthrocentesis on the improvement of internal derangement symptoms and jaw function in a series of patients with anterior disc displacement and closed lockjaw.

**Patients and methods:**

The study was based on a review of patients’ records before and after treatment using clinical examinations and radiographs. Visual analog scales were used to measure pain before and after arthrocentesis. Six patients (12 temporomandibular joints) with closed lock symptoms (2 cases) and internal derangements (4 cases) were treated at the Oswaldo Cruz Hospital. The mean follow-up was 11.5 months.

**Results:**

The mean maximum vertical opening before treatment was 31.83 mm and after arthrocentesis was 36.50 mm. The visual analog scale for pain before treatment was 7 points (mean) and after arthrocentesis the mean was 4.3.

**Conclusion:**

Arthrocentesis was shown to be effective in reducing pain and increasing jaw motion in this series of cases.

## INTRODUCTION

Temporomandibular joint (TMJ) arthrocentesis consists of lavage of the upper joint space of the TMJ done with no direct vision, aiming primarily to remove necrotic tissue, blood and pain mediators from the joint (Barkin, Weinberg, 2000).

Nitzan et al. (1991) first described TMJ arthrocentesis as the simplest form of surgery in the TMJ, aiming to release the articular disc and to remove adhesions between the disc surface and the mandibular fossa by means of hydraulic pressure from irrigation of the upper chamber of the TMJ.

Arthrocentesis has low morbidity, few risks and low cost compared to other TMJ surgical interventions, and may be conducted under local anesthesia in an outpatient clinic setting (Hasson, Levy, 1999; Carvajal, Laskin, 2000; Salazar et al., 2004).

Indications for arthrocentesis described in medical literature are: dislocation of the articular disc with or with no reduction, limitations of mouth opening originating in the joint, joint pain and other internal derangements of the TMJ (Nitzan, 1991; Frost, Kendell, 1999; Trieger et al., 1999; Yoda et al., 2002).

Clinical use of arthrocentesis in the TMJ is a new procedure among other possibilities of treating joint dysfunction. There is, therefore, a need for studies on the indications, success rate, and complications of this procedure.

This paper aims to present a series of patients undergoing arthrocentesis, to assess the results, and to provide a review of literature.

## MATERIAL AND METHODS

Six patients (twelve joints) presenting with pre-auricular pain, limited TMJ movements and closed lock were referred to the bucomaxillofacial surgery and trauma unit of the Oswaldo Cruz University Hospital in Recife, Pernambuco state.

All patients had undergone prior medical treatment for TMJ dysfunction (bite plates, muscle relaxants, compresses, diets and physical therapy) for at least 6 months with no clinical improvement. Four out of six patients had joint pain and functional limitation and two patients had closed lock (Table 1).

**Table 1:** Epidemiological aspects of participants.

Nº	Gender	Age	Main complaint	Diagnosis	Side
1	Fem	20	Pain	Internal derangement	Bilateral
2	Fem	34	Pain	Internal derangement	Bilateral
3	Fem	39	Pain	Closed lock	Bilateral
4	Fem	43	Pain	Internal derangement	Bilateral
5	Fem	39	Limited mouth opening	Closed lock	Bilateral
6	Fem	32	Pain	Internal derangement	Bilateral

Preoperative data included the clinical history, a physical exam and radiograms. This included progression time of TMJ dysfunction, the presence of facial asymmetry, unilateral or bilateral involvement, the amplitude of mandibular movements (maximum mouth opening, right and left laterality, and protrusion), the presence of joint noises, deviation on maximum mouth opening, and pain on mandibular movements, which was catalogued according to the pain visual analog scale. Radiography included a maxillary panoramic radiogram and a TMJ specific panoramic radiogram to establish anatomical changes of the mandibular condyle and reduced joint space.

**Figure 1:**
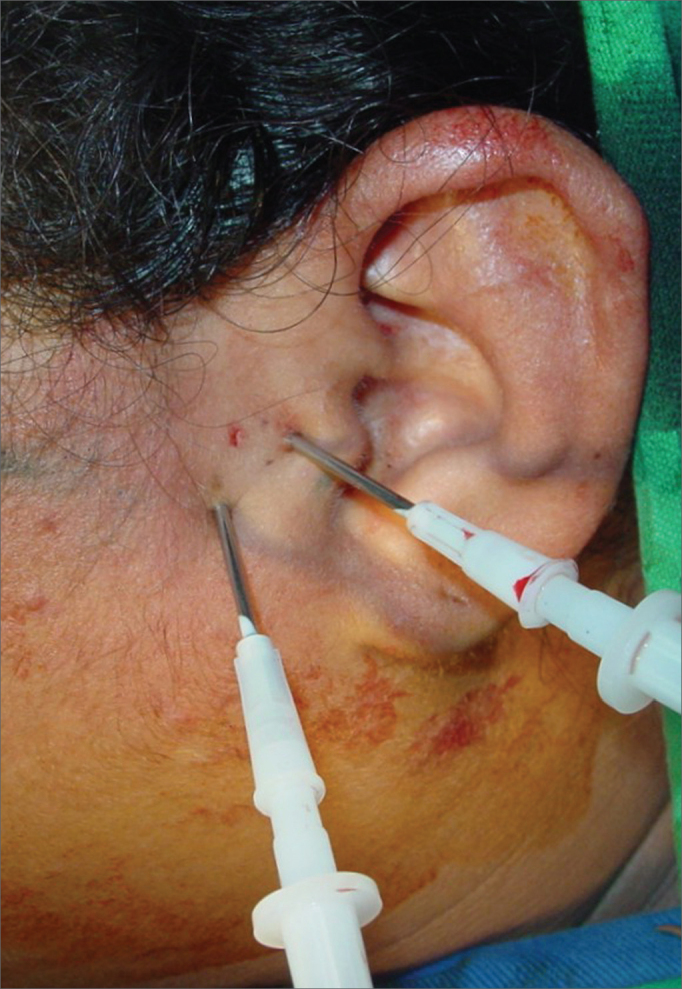
Clinical view of TMJ arthrocenthesis.

The first surgical procedure was TMJ arthrocentesis. All procedures were done by a single surgeon. Procedures were done under local anesthesia and sedation. Nitzan et al.'s (1991) surgical technique was used. A line was drawn from the corner of the eye - tragus and the first mark was made 10mm from the tragus and 0.5mm below the line. The second point was marked 20mm from the tragus and 1mm below the line. A 40×12 needle was placed on each point and the joint was irrigated with 250ml of saline under continuous pressure (Figure 1).

Postoperative follow-up for all patients was 11.5 months (6 to 17 months). Postoperative data were the same preoperative variables. This study was presented to and approved by the Pernambuco University Research Ethics Committee (number 004/04) and participants read and signed a free and informed consent form.

## RESULTS

### Objective findings

Table 2 summarizes and compares objective pre and postoperative findings. All patients had improved mouth opening following arthrocentesis. Preoperative mouth opening was 31.83mm ±8.10mm and postoperative mouth opening was 36.50mm ±6.89mm. Lateral movements and protrusion were unaltered. Further information is shown on Table 2.

**Table 2:** Measurement of the pre and postoperative mandibular movement amplitude.

Variable	Minimum	Maximum	Average	Mean	S.D.(1)
Maximum preop. mouth opening	20,00	42,00	31,83	30,50	8,10
Maximum postop. mouth opening	29,00	46,00	36,50	36,00	6,89
Preop. lateral movement to the right	3,00	8,00	6,00	6,50	1,78
Postop. lateral movement to the right	5,00	12,00	8,50	9,00	2,58
Preop. lateral movement to the left	4,00	9,00	6,58	6,25	1,74
Postop. lateral movement to the right	5,00	10,00	7,16	7,00	2,13
Preop. protrusion movement	3,00	10,00	7,16	8,00	2,71
Postop. protrusion movement	3,00	8,00	5,00	5,00	1,67

(1) - Standard Deviation

Three patients presented joint noises on the preoperative clinical exam. At the end of treatment, two no longer had joint noises and one had a reduction in joint clicks.

### Subjective findings

Every patient had moderate to severe preoperative pain. Preoperative pain visual analog scale scores averaged 7 ±1.78. On follow-up the pain score fell to an average 4.33 ±1.03. All patients reported an improved general clinical status and a reduction of internal derangement symptoms. (Table 3)

**Table 3:** Pre and postoperative pain scale scores.

Variable	Minimum	Maximum	Average	Mean	S.D.(1)
Preop. Pain Visual Analog Scale	5,00	10,00	7,00	6,50	1,78
Postop. Pain Visual Analog Scale	3,00	6,00	4,33	4,00	1,03

(1) - Standard Deviation

Maximum mouth opening, right lateral movements and the pain visual analog scale were statistically significant (p < 0.05) based on the Wilcoxon test. (Table 4)

**Table 4:** Statistical analysis of objective and subjective findings.

	Maximum postop. mouth opening	Postop. Pain Visual Analog Scale	Postop. Right Lateral Movement	Postop. Right Lateral Movement	Postop. mandibular protrusion
	-	-	-	-	-
	Maximum preop. mouth opening	Preop. Pain Visual Analog Scale	Preop. Right Lateral Movement	Preop. Right Lateral Movement	Preop. mandibular protrusion
Z	-2,023ª	-2,226^b^	-2,003ª	-7,36ª	-1,633^b^
Asymp. Sig. (2-tailed)	.043*	.026*	.045*	.461	.102

c. Wilcoxon Signed Ranks Test

a. Based on negative ranks

b. Based on positive ranks

## DISCUSSION

Arthrocentesis is the most recent surgical approach for internal derangement of the TMJ. In the past many cases of anterior displacement of the disc or closed lock that did not improve with medical treatment (bite plates, muscle relaxants, compresses, diet and physical therapy) were initially treated with surgical repositioning of the disc and arthroplasty of the mandibular fossa. Arthrocentesis has an intermediate place between the medical and the surgical forms of treatment (Salazar et al., 2004). Ease, lower cost of materials and excellent published results so far include this technique in the international protocol for the treatment of TMJ dysfunction (Spallacci aet al., 2000).

There are no longitudinal studies to compare the success and lack of success of this approach, and further studies are required to scientifically demonstrate the indication and predictability of results. Efficacy in various papers are: Murakami et al. (1995) - 70%; Dimitroulis et al. (1995) - 98%; Hosaka et al. (1996) - 79%; Fridrich et al. (1996) - 75%; Nitzan et al. (1997) - 95%. These studies suggest that arthrocentesis is an efficient method with relatively high success rates.

All patients had improvement in symptoms related to the intra-articular derangement and increased mandibular movements. These results are similar to published studies (Frost et al., 1992; Dimitroulis, 1995; Stein, 1995; Nitzan et al., 1997; Carvajal, Laskin, 2000). Internal derangement and closed lock conditions are usually associated with common symptoms such as pain, limited mouth opening and altered mandibular function, which may explain our results. Increased pain leads to reduced mouth opening and eventually to altered movements. By correcting one problem, the remaining two may also improve. (Carvajal; Laskin, 2000).

Lavage of the upper joint space reduces pain by removing inflammation mediators from the joint (Quinn, Bazan, 1990), increasing mandibular mobility by removing intra-articular adhesions (Spallacia et al., 2000), eliminating the negative pressure within the joint, recovering disc and fossa space (Nitzan et al., 1991), and improving disc mobility, which reduces the mechanical obstruction caused by the anterior position of the disc (Moses et al., 1989).

In our study we observed that symptom reduction resulted in improved mandibular function without necessarily producing an anatomical relationship between the mandibular head, the articular disc and the glenoid cavity. Rather, improved mandibular function appeared to be due to the removal of intra-articular adhesions and the resulting increased mobility of the disc.

## CONCLUSION

The methodology used in this series showed effective improvement in the treatment by arthrocentesis of patients with mandibular internal derangement and closed lock.
